# Serum Adiponectin and hsCRP Levels and Non-InvasiveRadiological Methods in the Early Diagnosis ofCardiovascular System Complications in Children andAdolescents with Type 1 Diabetes Mellitus

**DOI:** 10.4274/Jcrpe.1003

**Published:** 2013-09-18

**Authors:** Damla Gökşen, Ertürk Levent, Sakine Kar, Samim Özen, Şükran Darcan

**Affiliations:** 1 Ege University Department of Pediatric Endocrinology, School of Medicine, İzmir, Turkey; 2 Ege University, Department of Pediatric Cardiology, School of Medicine, İzmir, Turkey; 3 Ege University, Department of Pediatrics, School of Medicine, İzmir, Turkey

**Keywords:** type 1 diabetes mellitus, Adiponectin, C-reactive protein, carotid intima-media thickness, cardiovascular diseases

## Abstract

**Objective:** Adiponectin and high-sensitivity C-reactive protein (hsCRP) can be used as early biochemical markers of cardiovascular diseases (CVDs). Radiologically, non-invasive flow-mediated dilation (FMD) of the brachial artery and carotid intima-media thickness (CIMT) measurements may be used as indicators in the early diagnosis of CVDs. To compare the biochemical markers of atherosclerosis with radiological markers of CVDs (CIMT, FMD, ventricular systolic and diastolic functions) and to assess the relationship of these parameters with metabolic control in diabetic children and adolescents.

**Methods:** A total of 55 patients with type 1 diabetes mellitus (T1DM) of at least 5-year duration and 30 healthy subjects were included in the study. Serum adiponectin, hsCRP, hemoglobin A1c (HbA1c), and lipid levels were evaluated in the patients and in the controls. CIMT, FMD, ventricular systolic and diastolic functions were assessed by echocardiography.

**Results:** Mean age of the patients with diabetes was 17.6 years; mean diabetes duration was 10.4 years. Mean serum hsCRP was elevated in children with diabetes (0.21±0.31 vs. 0.10±0.16 μg/mL, p=0.00), while no significant difference from the controls was found in adiponectin levels. Mean CIMT was significantly higher in diabetic children compared to the control group (0.53±0.11 vs. 0.34±0.46 mm, p=0.00). Mean FMD of the diabetic children was significantly lower than that of the controls (6.86±2.85% vs. 12.13±1.99%, p=0.00). Diabetes duration was positively correlated with CIMT and negatively correlated with FMD. Right ventricular (RV) and left ventricular (LV) myocardial performance index (MPI) were higher in the patient group (p=0.00).

**Conclusions:** Our data suggest that in addition to standard echocardiography, tissue Doppler echocardiography, FMD, and CIMT can be used as early-stage radiological markers and hsCRP as an early-stage biochemical marker of atherosclerosis in the routine follow-up of T1DM patients.

**Conflict of interest:**None declared.

## INTRODUCTION

Early recognition of chron ic complications in cases with type 1 diabetes mellitus (T1DM) is very important for life expectancy and improvement of life quality (1,2). Cardiovascular disease (CVD) as a result of macrovascular atherosclerotic changes is the major cause of mortality among patients with diabetes mellitus, and these patients have 2-4 folds increased risk compared with non-diabetic subjects (3). Endothelial dysfunction is the main problem in the development of atherosclerosis, and it occurs in the early stage of T1DM, at a time atherosclerosis and micro- and macro-vascular complications of diabetes are still not clinically evident. Chronic inflammation has been shown to be the cause of the development and progression of endothelial dysfunction (4).

High-sensitivity C-reactive protein (hsCRP) is an acute-phase protein that is associated with systemic inflammation and has been shown to be increased in individuals with coronary artery disease (5,6,7,8). Adiponectin is a collagen-like protein secreted from the adipose tissue, and is believed to have an important role in hyperglycemia, dyslipidemia, and inflammatory mechanisms as well as in anti-atherogenic and anti-inflammatory effects (9,10). The reasons for the elevated adiponectin levels in persons with T1DM and for the paradoxical relationship between the known anti-atherogenic effects of adiponectin and premature mortality from coronary artery disease in T1DM are unclear.

CVD can be detected at an early stage before the symptoms become evident by non-invasive radiologic methods such as flow-mediated dilatation (FMD), carotid intima-media thickness (CIMT), and ventricular functions measurements, which may serve as indicators of endothelial dysfunction (11).

This study aimed to compare the biochemical markers of atherosclerosis, hsCRP, and adiponectin with early-stage radiological markers of CVD (CIMT, FMD, ventricular systolic and diastolic dysfunctions) and to assess the correlations of these parameters with metabolic control.

## METHODS

Fifty-five children and adolescents with T1DM (31 females and 24 males) with a disease duration of at least 5 years were recruited from the patient population attending the Department of Pediatric Endocrinology at Ege University School of Medicine. All of the patients were using basal-bolus insulin regimen. The patients were grouped according to duration of diabetes (Group 1: duration of 5-10 years and Group 2: duration longer than 10 years). In addition, 30 age-matched healthy subjects were recruited as control group (14 females and 16 males). Data on age, gender, duration of diabetes, insulin regimen, and daily requirement for insulin, and mean annual hemoglobin A1c (HbA1c) levels were collected from the medical records of the patients. Mean HbA1c levels of the preceding year were calculated. HbA1c measurements were performed by Nycocard II Reader (Axis-Shield Diagnostics Ltd, Dundee, UK) device via capillary method. Height was measured to the nearest centimeter using a rigid stadiometer. Weight was measured unclothed to the nearest 0.1 kg using a calibrated balance scale. Body mass index (BMI) was calculated using the weight (kg)/height (m2) equation. Standard deviation scores (SDS) for weight, height and BMI were calculated using the reference values for Turkish children (12). Measurements of blood pressure were performed in all cases after a period of resting and were repeated at least 3 times with 10-minute intervals. Subjects with systolic and/or diastolic blood pressure above the 95th percentile were accepted as hypertensive (13). Pubertal status of each case was defined according to Tanner criteria. Fasting blood glucose, lipid profiles, and hsCRP were measured by an automatic analyzer (Siemens Healthcare Diagnostic Ltd, Newark, DE,USA) in both patient and control groups. Serum samples for adiponectin were stored at -70°C after centrifugation. Adiponectin levels were measured by ELISA method, using a human adiponectin ELISA kit (BioVendor®, Czech Republic).

**Echocardiographic Examinations**

A complete echocardiographic evaluation by a pediatric cardiologist who was blinded to the diagnoses was performed in all subjects with a two dimensional, M-mode and Doppler echocardiogram using a Vivid 7 system with a 3 MHz transducer (GE Vinmed, Ultrasound AS, Horten, Norway). All subjects were kept in the left decubitus position during the examination. Measurements of shortening fraction (FS) and the ejection fraction (EF) were obtained from M-mode echocardiographic tracings with 2D imaging. The measurements were made using standard techniques in accordance with the recommendations of the American Society of Echocardiography (14).

Right and left ventricular myocardial performance indices (RVMPI and LVMPI) and inflow velocities (E-wave and A-wave velocities) were obtained in the four-chamber plane with a pulsed-wave (PW) Doppler. Mean values for these indices obtained from 5 consecutive cardiac cycles were used in the analysis. The details of these measurements were given in a previous study (15).

Tissue Doppler (TD) evaluation was obtained with the sample volume placed at the lateral corner of the mitral, tricuspid annulus and, subsequently, on the medial corner from the apical four chamber view. In each region, systolic (S) wave, early diastolic (Ea), and late diastolic (Aa) velocities and ejection time (ET) were recorded. Also, the isovolumetric contraction time (ICT) and the isovolumetric relaxation time (IRT) were measured from the end of the mitral annular velocity pattern to the onset of the S-wave and from the end of the S-wave to the onset of the mitral annular velocity pattern.

**Carotid Intima-Media Thickness (CIMT) Measurements**

High-resolution B-mode ultrasonography was performed in all patients on the right carotid artery using the same echocardiography device with a 12 MHz high-resolution linear probe. The patients were positioned to have their heads turned slightly to the left side. After the carotid artery was measured at the end-diastole and all measurements were recorded, the CIMT was calculated by taking the mean of all three measurements (15,16).

**Brachial Artery (FMD) Measurements**

After the patients had rested for a short while, their right arm was fixed at the extension position. The location where the best image of the brachial artery could be obtained (2-5 cm above the cubital region) was marked with the 12 MHz probe, and the probe was fixed. All measurements were performed at the internal diameter of the brachial artery at the end-diastole and were recorded. After the optimal basal measurement, systolic pressure was increased to 250 mmHg with the help of the cuff placed on the upper arm, and the ischemia was sustained for 5 minutes. After the cuff was deflated, brachial artery measurements were repeated at 60-second intervals. Basal measurement and brachial artery dilatation after the ischemia were calculated as FMD% (17).

**Statistical Analysis**

SPSS (Statistical Package for Social Sciences) for Windows version 18.0 package program was used in the evaluation of data. Results of parameters for control and patient groups were given as standard deviations. Student’s t-test, Mann-Whitney U-test, Fisher’s exact test, and chi square test were used to compare data in patient and control groups. Correlation between parameters was investigated by Spearman correlation analysis. Level of significance was set at p<0.05.

The study was approved by the Local Ethics Committee of the Medical School of Ege University (10.09.2010 - 10-8/3). Informed consent was taken from the families of all patients. 

## RESULTS

Clinical characteristics of the patient and control groups are shown in Table 1. The two groups did not differ regarding age, gender, BMI, height, weight, and pubertal stage. There was no significant difference between the two groups in terms of gender (p=0.39). All patients were pubertal, and there was no difference in pubertal stage between the two groups. Mean diabetes duration and mean age were 10.2±3.8 and 17.6±4.0 years, respectively, in the patient group. Mean diabetes duration was 7.28±1.24 years in Group 1 patients and 13.5±2.89 years in Group 2 patients. There was no statistically significant difference in systolic and diastolic blood pressures between the patients and controls (p=0.2 and p=0.7).

Mean HbA1c value was 7.9±1.6% in the patients. Mean HbA1c value in the preceding year was 8.0±1.2% in Group 1 and 7.6±1.1% in Group 2 (p=0.16). There also were no differences in lipid and adiponectin levels between the patients and the controls. Mean hsCRP levels were significantly increased in the patient group (0.21±0.31 mg/dL vs. 0.10±0.16 mg/dL) (p=0.00). Laboratory data in the patient and control groups are given in Table 2. There was a positive correlation of hsCRP with diabetes duration, LDL, and BMI (r=0.341, p=0.01; r=0.380, p=0.00; r=0.312, p=0.02, respectively). Also, hsCRP levels were positively correlated with BMI in the control group (r=0.355, p=0.02). Serum adiponectin levels in Group 1 and Group 2 were 14.6±6.32 µg/mL and 15.9±6.02µg/mL, respectively (p=0.45). No correlation was found between adiponectin levels and either diabetes duration (r=0.66; p=0.63) or mean HbA1c of the preceding year (r=0.015; p=0.91). Adiponectin levels were negatively correlated with BMI in both patient and control groups (r=-0.347, p=0.00, r=-0.298, p=0.02, respectively). There was no correlation between hsCRP and adiponectin level (r=0.11; p=0.40). HDL cholesterol was significantly decreased, and daily insulin doses were significantly increased in the poorly controlled group. There was no difference in adiponectin (p=0.60) and hsCRP levels (p=0.56) according to metabolic control (Table 3).

**Ventricular Function**

RVMPI and LVMPI values were higher in the patient group (p=0.00) (Table 4). Higher Mean RVMPI and LVMPI were obtained in Group 2 compared to Group 1 (0.41±0.11 and 0.34±0.12) (p=0.03), (0.45±0.10 and 0.38±0.09) (p=0.00). There was a positive correlation of diabetes duration with RVMPI and LVMPI (r=0.306, p=0.02 and r=0.394, p=0.00; respectively).

**Diastolic Function**

Mean tricuspid A-wave value in the patient group was higher, whereas E/A ratio was lower (for both p=0.00) (Table 4). No significant difference was found in mean E, A, E/A, and deceleration time (DT) values between Groups 1 and 2 (p=0.09, p=0.36, p=0.94, p=0.56) (Table 5). Additionally, Ea/Aa was significantly lower (p=0.00) and tricuspid S value was significantly higher (p=0.02) in the patient group. While mitral Am, Em/Am, and DT values, which were obtained from left ventricular diastolic functions in TD evaluation, were similar between the patient and control groups, mitral Sm and Em values in the patient group were significantly higher than the values in the control group (p=0.00 and p=0.01). No correlation was found between metabolic control, diabetes duration and diastolic function.

**Carotis Intima-Media Thickness (CIMT)**

Mean CIMT in the patient group was higher than the control group (p=0.00) (Table 4). Mean CIMT value in Group 2 was higher than in Group 1 (0.57±0.10 and 0.5±0.12; p=0.02) (Table 5). CIMT increased as diabetes duration increased (r=0.4, p=0.000). CIMT did not correlate with metabolic control, lipid profile, hsCRP, and adiponectin levels.

**Flow-Mediated Dilatation (FMD)**

Mean FMD percent value was lower in the patient group than in the control group (p=0.00). Mean FMD value was lower in Group 2 (p=0.01) (Table 5). There was a negative correlation between FMD percent value and diabetes duration (r=-0.510, p=0.00). There was a negative correlation between FMD percent value and both adiponectin and hsCRP levels in the patient group (for adiponectin r=- 0.278, p=0.04; and for hsCRP r=-286, p=0.03). No correlation was found between mean FMD percent value and either metabolic control or lipid levels (r=-0.06, p=0.65 and r=-0.05, p=0.7; respectively).

There was a negative correlation between FMD and RVMPI (r=-0.393; p=0.00). 

## DISCUSSION

In T1DM, for prevention and early diagnosis of macrovascular complications, it is very important to understand the pathophysiology of CVD. Studies in this issue are still ongoing (18,19). In our study, a number of markers of subclinical CVD and of cardiac (LV and RV) function were simultaneously examined in a population of asymptomatic children and adolescents with T1DM receiving a basal-bolus regimen. With conventional and TD echocardiography, we have shown the development of diastolic dysfunction in both ventricles alongside with an increased CIMT and a decreased FMD% in the course of T1DM.

Adiponectin, which is synthesized in the adipose tissue, is known to have anti-atherogenic, anti-inflammatory, and insulin-sensitizing effects (10,20). In some studies, adiponectin levels have been shown to be higher in children and adolescents with T1DM than in normal controls (21,22,23). In our study, serum adiponectin levels in children and adolescents with T1DM were comparable to those in the control group. Similar to our results, Morales et al (24) reported no significant difference in serum adiponectin levels between T1DM patients and a healthy control group. The causes of the differences among the different studies are not clear, but they may be attributed to differences in ages at diagnosis, genetic factors, ethnic backgrounds (different HLA values, autoantibody frequency, etc.). In some studies, a correlation was reported between diabetes duration and serum adiponectin level (23,24). Lindström et al (25) reported that serum adiponectin level was higher in the group with longer duration (>10 years). This high adiponectin level was attributed to renal function deterioration related to diabetes duration. In our study, no difference in adiponectin levels was found according to diabetes duration. There was also no correlation between metabolic control and adiponectin levels (p=0.60). In a recent meta-analyses, it was also found that adiponectin levels and CVD are not correlated (26,27,28).

Although there was no proven correlation between increased hsCRP levels and endothelial dysfunction in adult T1DM patients (29), this relationship is not clear in children with T1DM. MacKenzie et al (30) could not show any correlation between hsCRP and endothelial dysfunction in their study in children and adolescents with T1DM. In our study, serum hsCRP levels in the patient group were significantly higher than those in the control group, and there was a negative correlation between hsCRP and FMD%. Atabek et al (31) reported that CRP was positively correlated with CIMT and that CRP was also associated with common carotid artery (CCA) structure and functions in children and adolescents with T1DM. In our study, a positive correlation between hsCRP and BMI and a negative correlation between hsCRP and FMD were found. However, we did not find any correlation between hsCRP and CIMT, another radiological marker of atherosclerosis. We can conclude that concomitance of obesity and T1DM may cause a bigger inflammatory reaction than that observed in children with with normal BMIs. Therefore, it is very important that in children and adolescents with T1DM, BMI should be within normal limits and, if necessary, weight decreasing programs should be conducted to prevent vascular complications. In some studies, correlations have been reported between cholesterol (6), diabetes duration, age, and glycemic control (7). While there was no correlation between glycemic control and hsCRP in our study, a positive correlation was found between serum LDL, diabetes duration and hsCRP. hsCRP, a marker for coronary artery disease, was reported to negatively correlate with adiponectin levels in subcutaneous fat tissue in patients with coronary artery disease (7). No correlation was detected between serum hsCRP and adiponectin levels in our study.

In a prospective longitudinal study, it has been detected that serum adiponectin level was a negative marker of coronary artery disease in patients with T1DM (32). In our study, although no correlation was detected between adiponectin level, which is known to have an anti-atherogenic effect, and CIMT, which is an early radiological marker of atherosclerosis, a negative correlation was detected between adiponectin level and FMD, which is a marker of endothelial dysfunction, and therefore, of atherosclerosis.

CIMT, FMD, arterial stiffness, ventricular systolic and diastolic function measurements are non-invasive US methods used to reveal cardiovascular problems in patients with T1DM (33,34,35). With conventional and TD echocardiography, we have shown normal systolic function and decreased diastolic dysfunction in both ventricles in our T1DM patients. However, we have not found any correlations between systolic and diastolic functions and DM duration, metabolic control, adiponectin level, and hsCRP. Suys et al (36) demonstrated marked filling abnormalities in the left ventricles, partial filling abnormalities in the right ventricles, more atrial dependency for ventricular filling, and low E/A ratio in T1DM patients who were not hypertensive and who were aged between 4 and 22 years. They also were not able to show any correlation between metabolic control and ventricular function, a finding which is similar to our results. Diastolic and systolic functions were reported to be correlated with metabolic control in studies performed on adults (37,38).

In our study, CIMT measurement which is considered a surrogate marker of subclinical atherosclerosis was found to be increased in T1DM cases and showed a positive correlation with diabetes duration and a negative correlation with FMD% which is also an endothelial function marker. There was no correlation between CIMT and either serum adiponectin or hsCRP levels. In contrast to our study, Heilman et al (39) reported that CIMT was markedly high in 30 diabetic children with a mean age of 4.7±18.6 years and that it showed a positive correlation with HbA1c, age, and systolic blood pressure. Jarvisalo et al (11) and Dalla Pozza et al (40) reported presence of signs of subclinical atherosclerosis in their patients although their diabetes duration was less than 5 years. In our study also, signs of subclinical atherosclerosis were more prominent in patients even in the early stages of T1DM when compared with healthy controls. CIMT increased as diabetes duration increased, and the increase in CIMT was accompanied by endothelial dysfunction.

Endothelial dysfunction which develops prior to the emergence of structural and clinical signs of atherosclerosis, is believed to play a key role in atherogenesis. It has been shown that endothelial dysfunction occurs early in diabetic children and may appear before the increase in CIMT (11,41,42). Donaghue et al (43), in 20 adolescent patients with diabetes, have shown decreased endothelium and smooth muscle functions. Wiltshire et al (44), in 36 diabetic children with a mean age of 14 years and mean diabetes duration of < 6 years, has shown decreased FMD% without any correlation with diabetes duration and glycemic control. In our study, FMD% was significantly decreased in the diabetic group and this finding was correlated with duration of diabetes but not with metabolic control. These results indicate that despite good metabolic control, endothelial dysfunction can start at an early stage, and becomes more prominent with time.

Shivalkar et al (45), in 100 adult diabetic patients with a disease duration of 2-36 years, observed the appearance of abnormal FMD within the first decade of the disease as well as segmental left- and right-sided cardiac diastolic and systolic dysfunction, compared with age-matched controls. In this same study, both right and left ventricular diastolic dysfunction and a milder rate of left ventricular systolic dysfunction were detected. In our study, we detected ventricular diastolic function disorder particularly in the right ventricle. Systolic functions were preserved, and there was a negative correlation between FMD and only the RVMPI.

In conclusion, our study supports the hypothesis that endothelial dysfunction and atherosclerosis start at the early stage of the disease in children and adolescents with T1DM. Although the markers indicating endothelial dysfunction are not directly related to metabolic control, their severity is increased as the disease duration increases. For the early diagnosis of CVD in children and adolescents with T1DM, hsCRP can be used as a biochemical marker, and CIMT, FMD, RVMPI measurements can be used for ultrasonographic evaluation.

Our study has several limitations. First, the small sample size of the cohort may not be enough to derive a conclusion and make recommendations on routine cardiovascular evaluation of T1DM patients. Longitudinal studies are needed to show whether pulsed-wave Doppler or TD echocardiography should be used for cardiovascular evaluation. However, our data suggest that in addition to standard echocardiographic methods, TD echocardiography, FMD, and CIMT can be performed, and HsCRP can be used as a biochemical marker of atherosclerosis in the routine follow-up of T1DM patients.

**Acknowledgements**

This study is sponsored by the Department of Research Project of Ege University.

## Figures and Tables

**Table 1:**
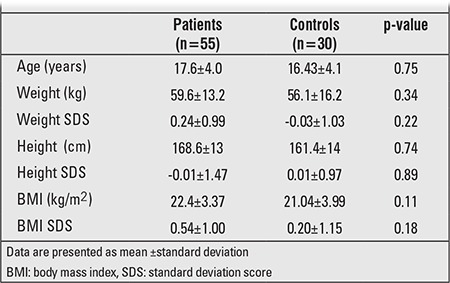
Demographic characteristics of the patient and control groups

**Table 2:**
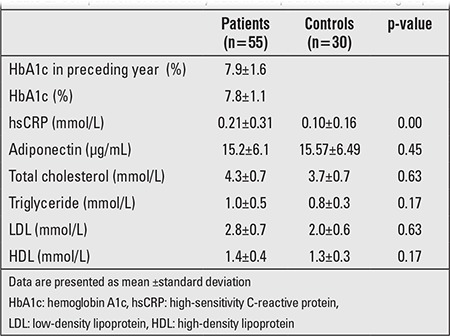
Comparison of laboratory data in the patient and control groups

**Table 3:**
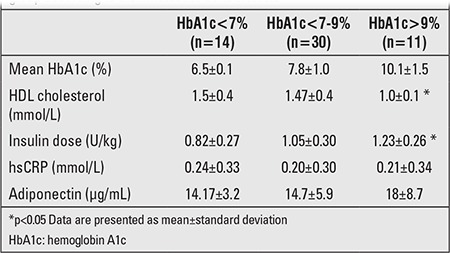
Daily insulin dose, serum high-density lipoprotein (HDL), high-sensitivity C-reactive protein (hsCRP), and adiponectin levels in the patientgroup according to their metabolic control levels

**Table 4:**
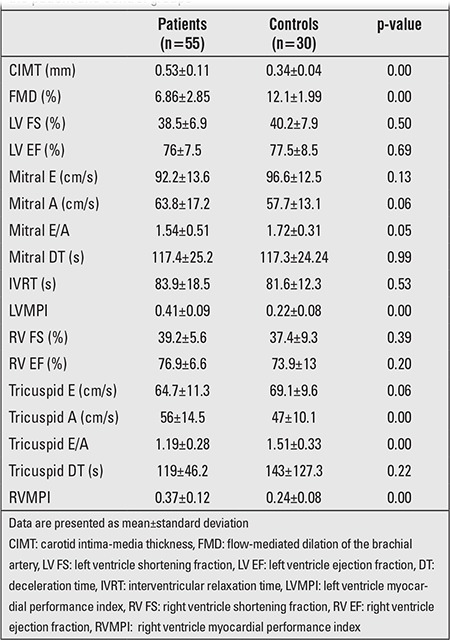
Pulsed-wave Doppler and B- mode echocardiographic data inthe patient and control groups

**Table 5:**
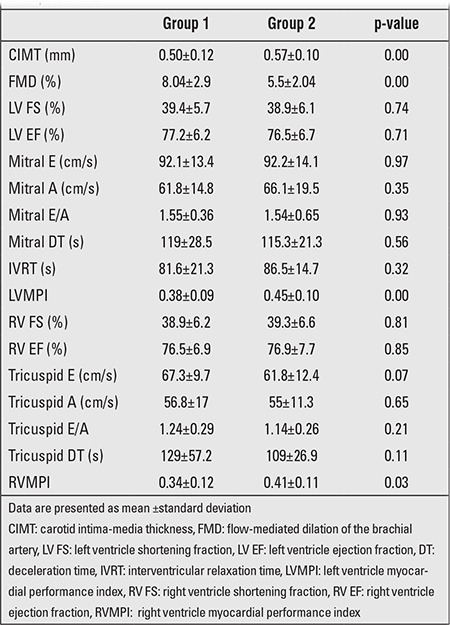
Pulsed-wave Doppler and B-mode echocardiographic data inGroups 1 and 2
